# Medical specialists’ use and opinion of video consultation in Denmark: a survey study

**DOI:** 10.1186/s12913-024-10868-6

**Published:** 2024-04-24

**Authors:** Olivia Mandal Møller, Sif Sofie Vange, Anne Sofie Borsch, Tomas Norman Dam, Anja MB Jensen, Signe Smith Jervelund

**Affiliations:** 1 Section for Health Services Research, Department of Public Health, University of Copenhagen, Øster Farimagsgade 5, 1353 Copenhagen, Denmark; 2 Dermatology Clinic, 4800 Nykøbing F, Denmark

**Keywords:** Telemedicine, Video consultation, Remote consultation, Medical specialty, Medical specialist, Secondary care, Attitudes, Acceptability of healthcare, Suitability, Denmark

## Abstract

**Background:**

The COVID-19 pandemic accelerated the use of telemedicine which is seen as a possibility to reduce the pressure on healthcare systems globally. However, little research has been carried out on video as a consultation medium in medical specialists’ practice. This study investigated the use of and opinion on video consultation among specialists in Denmark.

**Methods:**

An online survey on use of video consultation, as well as relevance of and opinion on video consultation, was distributed to all 963 medical specialists in private practice in Denmark throughout May and June 2022, resulting in 499 complete answers (response rate: 51.8%). Data were analysed using descriptive and logistic regression analyses, and data from open text fields were analysed using thematic network analysis.

**Results:**

Among the respondents, 62.2% had never used video consultation, while 23.4% were currently using video consultation, most particularly among psychiatrists. A total of 47.3% found video consultation medically irrelevant to their specialty, especially radiologists, plastic surgeons, ophthalmologists and otorhinolaryngologists. According to the specialists, video consultation was most suitable for follow-up consultations and simple medical issues, where the patient had an established diagnosis. In these cases, mutual trust remained present in video consultations. Better access for the patients and fewer cancellations, especially for psychiatrists, were highlighted as benefits. IT problems were reported as obstacles hindering optimal use of video consultation.

**Conclusion:**

The political aspiration to digitization in healthcare systems should be rooted in professionals’ and patients’ perceptions and experiences with video consultation which emphasize that it is not a standard tool for all consultations.

**Supplementary Information:**

The online version contains supplementary material available at 10.1186/s12913-024-10868-6.

## Background

Telemedicine is generally seen by healthcare systems worldwide as a promising mean to reduce the pressure on the healthcare systems [1]. Video consultation in the clinical setting has been widely implemented in the European countries, especially the UK [2–4], but has not previously been an integrated element in the Danish healthcare system. International studies have primarily investigated video consultation in the context of general practitioners (GP), ambulatories, and hospitals, and prior findings in terms of effectiveness and accessibility to care are largely positive although technological challenges are continuously highlighted [3–5]. Successful use depends on patients’ and clinicians’ willingness and acceptance towards new technologies, technological training and support as well as patients’ specific medical conditions [2–4, 6]. Clinicians appreciate greater flexibility and saved time for their patients. However, they express concerns about their own workloads, and their ability to make accurate clinical decisions by video, citing difficulties in establishing rapport and detecting nonverbal cues, which in a physical setting contribute to the overall disease picture [2, 7–9]. Less attention has been given to medical specialists in former research.

Medical specialists practising in non-hospital settings serve as the medical experts in the realm of medical diagnostics, treatments, and patient monitoring. These specialists engage with the individual patient on a limited basis, typically conducting one or two consultations, unless dealing with individuals with chronic conditions, who are commonly subjected to extended periods of care. Thus, it is crucial for specialists to have optimal conditions for their medical practice in their clinical encounter. Studies exploring the use of video consultation in medical specialists’ practice have shown that the use of video consultation for patients with chronic diseases [6] and in follow-up consultations [10] can be appropriate. Within psychiatry, a review showed that video consultation for elderly patients with depression worked well [11], while another study based on the perspective of psychiatrists highlighted that handling certain topics such as traumatic incidents was not appropriate by video [12]. Among medical specialists, there is wide variation in the type and premise of consultations, including the size of the role that dialogue, vision and medical tactile interaction, and we lack knowledge on specialty-specific conditions for and attitudes to video consultation.

**Fig. 1:**
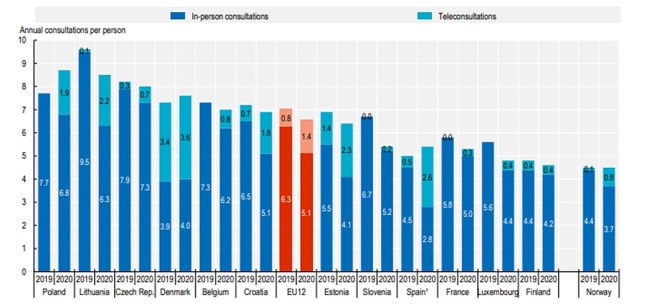
Number of teleconsultations and physical consultations with an unspecified doctor in 2019–2020 from OECD/European Union (2022), *Health at a Glance: Europe 2022: State of Health in the EU Cycle* [15]

In 2018, before the COVID-19 pandemic, a digitization strategy for the Danish public healthcare system was launched [13] aiming to make every third consultation virtual [14]. The Danish healthcare system is known for its advanced use of digitalization and had the highest share of teleconsultations (including online and telephone consultations) among all EU countries before the pandemic (Figs. 1, [15]) using the “My Doctor” app for video consultation options [16].

The COVID-19 pandemic accelerated the use of video consultation both in the Danish healthcare system [17] and in other countries [18, 19], to ensure essential activity while reducing the risk of viral transmission. In 2020, at the expense of physical consultations, which fell by almost 20% on average across EU countries, tele-consultations (including online and telephone consultations) increased in many EU-countries, especially in Poland, Denmark and Spain [15]. In Denmark, from March to December 2020, 42,748 consultations were conducted by video in specialist practice [20] out of a total of approximately 5,500,000 consultations in that same year [21]. The Danish healthcare system is universal and financed by general taxation, and access to general and specialized medical practitioners and hospitals is free of charge [22]. Access to medical specialists requires a referral from a GP, except for ophthalmologists and otorhinolaryngologists, which can be visited directly. Medical specialists are private practitioners, but are contracted to the five Danish regions through a fee-for-service-agreement [22]. Following the COVID-19 pandemic, video consultation has become a permanent feature within the collective agreement for medical specialists from 1 April, 2022, stating that video consultations should be offered by all specialists in private practice as long as they deem it relevant for their specialty and upon patient request [23]. While numbers for general practice in the first six months in 2022 revealed that 1.1% of all consultations were conducted by video [24], similar numbers are not available for specialist practice.

There is limited knowledge on how much medical specialists make use of video consultation overall and within their specialty, whether they find it relevant to their specialty and their overall attitudes towards video consultation. Insights into how and why different medical specialties use video consultation, as well as the particular conditions under which medical specialists consider video consultation relevant and suitable, constitute crucial knowledge for future planning of health services. Thus, the aim of this study was to map the use of and opinion on suitability of video consultation as well as the experience of care and trust in video consultations among medical specialists in private practice in Denmark with a special focus on the role of specialty.

## Methods

### Study design and setting

The study was based on a nationwide survey, to be completed anonymously, and was distributed by email in May 2022 through the Association of Medical Specialists to all 963 medical specialists in private practice covering 15 specialties in Denmark. A reminder was sent one week later.

### Description of survey instrument

Development of the survey was based on reviewing relevant literature exploring GPs’ and outpatient services’ use of video consultation, and on reports from the Danish health authorities. In addition, we identified and included relevant topics based on insights into video consultation acquired through nine qualitative interviews with medical specialists and through telephone conversations with medical specialists and their secretaries. To qualify the survey, we pilot tested it among a medical specialist before distribution.

The survey consisted of between five and 12 questions (see Additional file 1 for an English translation of the survey)– depending on responses to previous questions– and took approximately three to seven minutes to complete. The items covered specialty, region, current and previous use of video consultation, as well as assessment of relevance and opinion of video consultation. Open text fields were available for several of the questions, enabling elaboration of the responses.

To obtain knowledge on the use of video consultation, we posed the question: ‘Have you used or do you use video consultations in your clinic?’ with the response options listed in Table 1. Current users were identified by combining three of the response categories. We investigated the specialists’ opinion on video consultation becoming part of their collective agreement through the question: ‘What is your opinion on video consultation becoming part of the collective agreement in 2022?’ with the response options on a 5-point Likert scale. (1) ‘Positive’, (2) ‘Mostly positive’, (3) ‘Neutral’, (4) ‘Mostly negative’ and (5) ‘Negative’. We combined ‘positive’ and ‘mostly positive’ as well as ‘negative’ and ‘mostly negative’. An open text field was available.

The experiences of video consultation amongst those who have previously used or currently use video consultation were explored through the question: ‘What are your experiences with video consultations?’ with the response options (1) ‘Very positive’, (2) ‘Positive’, (3) ‘Neutral’, (4) ‘Negative’ and (5) ‘Very negative’. ‘Positive’ and ‘very positive’ were combined, as were ‘negative’ and ‘very negative’. An open text field was also available.

To understand the suitability of video consultation, we included a question regarding types of consultations for which video consultation is appropriate, such as anamnesis, treatment, follow-up, and so on (see Table 2). Multiple responses and elaboration were possible. Our survey asked: ‘To what degree would you offer video consultation if the patient requested it?’ with the response options depicted in Table 3 to obtain information on medical specialists’ willingness to offer video consultation and for which specialties video consultation is not considered medically relevant. As a follow-up to the ‘medically irrelevant’ option, we asked the respondents to specify why video consultation was not relevant to them, and suggested categories such as the medical issues not being suitable for video, conversation by video complicating an optimal consultation, and so on. Elaboration was possible.

Lastly, the respondents answered: ‘How do you experience care and trust in the relationship with the patient during a video consultation compared to a physical consultation?’ Response options were (1) ‘Much better’, (2) ‘Better’, (3) ‘The same’, (4) ‘Worse’ and (5) ‘Much worse’. We combined ‘better’ and ‘much better’ as well as ‘worse’ and ‘much worse’. Again, elaboration was possible.

### Statistical and open text analysis

Using descriptive statistics, we described and summarized the data. To compare unadjusted proportions of outcome variables by medical specialty, we performed Chi^2^-tests with 95% CIs. We used binary logistic analysis to examine the association between medical specialty and medical relevancy of video consultation. SPSS version 28 was used for data analysis.

We analysed comments from open text fields using thematic network analysis by systematically coding the comments and identifying themes, which were further elaborated and summarised e.g., *suitability of video consultations, lack of holistic view, trust, time and accessibility* [25]. We selected relevant quotations to support and nuance the quantitative data.

### Ethical considerations

Following Danish legislation, we registered the study at the Faculty of Health and Medical Sciences at the University of Copenhagen. The processing of personal data in the study was approved by the Faculty of Health and Medical Sciences, University of Copenhagen, and data were handled according to GDPR. According to Danish legislation, surveys which do not include human biological material should not be reported to the National Scientific Ethics Committee [26]. However, since the overall project also encompasses a focus on vulnerable population groups, such as individuals with psychiatric disorders, we aimed to ensure that the overall project was compliant with relevant Danish and international standards and guidelines for research ethics. As a result, we submitted an application of the overall project to the Research Ethics Committee for the Faculty of Science & Faculty of Health and Medical Sciences, University of Copenhagen. The committee approved our project. Respondents were informed about the project in the invitation email and consented to participate by completing the survey. Thus, informed consent was obtained from all respondents. To avoid identification of individual responses, we carefully avoided separating survey data into small categories.

## Results

A total of 519 responded to the survey. We excluded 20 incomplete responses, resulting in 499 respondents and a sample representing 51.8% of medical specialists in private practice in Denmark. The variance of non-respondents between specialties was small with a response rate around 50% for most of the specialties. The lowest response rate was found among child and adolescent psychiatrists (36.8%) and the highest among plastic surgeons (73.3%).

### Use of video consultations

The distribution of previous and current use of video consultation by specialty is presented in Table 1. Among the respondents, 62.2% had never used video consultation, 14% used it only during COVID-19 lockdown and 23.4% were currently using video consultation. The highest proportion of current users were psychiatrists (84.7%) followed by child and adolescent psychiatrist (71.4%) and neurologists (62.2%). Video consultation was not used at all in radiology, surgery and otorhinolaryngology. Of the medical specialists currently using video consultation, video consultation made up 0–10% of all consultations for the majority (70.1%), while 17.9% used it in 11–20% of their consultations and 12% used it in 21% or more of their consultations, with psychiatrists using it the most (see Additional file 2).

**Table 1:** Use of video consultation, by medical specialty. *(N = 499*)

	Has never used video consultation	Used video consultation during COVID-19 lockdown and does not use it anymore	Current users			Total
			Used video consultation before and during COVID-19 lockdown and still uses it	Used video consultation during COVID-19 lockdown and still uses it	Used video consultation after the new collective agreement took effect on 1 April 2022	
	n (%)	n (%)	n (%)	n (%)	n (%)	n (%)
Specialty						
Total	312 (62.2)	70 (14)	117 (23.4)			499 (100)
			19 (3.8)	87 (17.4)	11 (2.2)	
Dermato-venerology	18 (34)	20 (37.7)	15 (28.3)			53 (100)
			1 (1.9)	12 (22.6)	2 (3.8)	
Neurology	5 (31.3)	1 (6.3)	10 (62.2)			16 (100)
			0	9 (56.3)	1 (6.3)	
Psychiatry	5 (7.7)	5 (7.7)	55 (84.7)			65 (100)
			8 (12.3)	43 (66.2)	4 (6.2)	
Anaesthesiology	10 (71.4)	0	4 (28.5)			14 (100)
			2 (14.3)	1 (7.1)	1 (7.1)	
Child and adolescent psychiatry	0	2 (28.6)	5 (71.4)			7 (100)
			0	5 (71.4)	0	
Radiology	9 (100)	0	0			9 (100)
			0	0	0	
Gynaecology and obstetrics	37 (80.4)	3 (6.5)	6 (13)			46 (100)
			2 (4.3)	3 (6.5)	1 (2.2)	
Internal medicine	17 (70.8)	4 (16.7)	3 (12.5)			24 (100)
			0	3 (12.5)	0	
Surgery	24 (92.3)	2 (7.7)	0			26 (100)
			0	0	0	
Orthopaedic surgery	15 (88.2)	1 (5.9)	1 (5.9)			17 (100)
			0	0	1 (5.9)	
Plastic surgery	12 (85.7)	1 (7.1)	1 (7.1)			14 (100)
			0	1 (7.1)	0	
Paediatrics	2 (11.8)	6 (35.3)	9 (53)			17 (100)
			1 (5.9)	8 (47.1)	0	
Rheumatology	13 (52)	6 (24)	6 (24)			25 (100)
			3 (12)	2 (8)	1 (4)	
Ophthalmology	65 (83.3)	11 (14.1)	2 (2.6)			78 (100)
			2 (2.6)	0	0	
Otorhinolaryngology	80 (90.9)	8 (9.1)	0			88 (100)
			0	0	0	

### Opinion on and experiences with video consultation

Of current users, 79.5% found video consultation as a permanent option positive, while the same was true for 22.8% of the nonusers (*p* < 0.001) (see Additional file 3). From the open text field, it appeared that the negative experiences often related to technical difficulties such as setting up the IT system and poor video quality. Moreover, some found that clinical evaluations were difficult to assess through the screen and as a result some respondents concluded that video consultation had to be followed up by a physical consultation. In contrast, some specialists reported that if the technology worked, it was possible to conduct consultations which were as sufficient as physical consultations but that video consultations could be used in combination with physical consultations.

### Suitability according to consultation type

Table 2 shows which type of consultations were considered suitable for video consultation among the medical specialists who at some point have used video consultation. Overall, video consultation was found suitable for follow-up of simple medical issues or for patients with an established diagnosis. More specialists preferred to use video consultation in longer treatment courses (40.6%) compared to shorter treatment courses (28.9%), and more specialists preferred to use video consultation with a known patient (64.7%) compared with a new patient (16.6%). Consultations on follow-up in general (59.4%), medication control (43.9%) and providing follow-up test results (45.5%) were also rated as suitable for video consultation. Of the specialists who had used video consultation at some point, 19.3% stated that video consultations were not suitable for any types of consultations.

**Table 2:** Types of consultations suitable for video consultation, by medical specialty. Only medical specialists who at some point have used video consultation. *(N = 187)*

	Short treatment courses	Long treatment courses	Known patient	New patient	Anamnesis	Follow-up of test result(s)	Objective assessment	Medication control	Treatment	Follow-up consultations	No suitable types of consultations	Total
	n (%)	n (%)	n (%)	n (%)	n (%)	n (%)	n (%)	n (%)	n (%)	n (%)	n (%)	n
Specialty												
Total	54 (28.9)	76 (40.6)	121 (64.7)	31 (16.6)	45 (24.1)	85 (45.5)	8 (4.3)	82 (43.9)	47 (25.1)	111 (59.4)	36 (19.3)	187
Dermato- venerology	6 (17.1)	14 (40)	24 (68.6)	3 (8.6)	5 (14.3)	9 (25.7)	2 (5.7)	16 (45.7)	6 (17.1)	20 (57.1)	7 (20)	35
Neurology	4 (36.4)	4 (36.4)	8 (72.7)	0 (0)	1 (9.1)	7 (63.6)	0 (0)	5 (45.5)	2 (18.2)	9 (81.8)	2 (18.2)	11
Psychiatry	26 (43.3)	34 (56.7)	54 (90)	14 (23.3)	19 (31.7)	31 (51.7)	3 (5)	35 (58.3)	30 (50)	40 (66.7)	5 (8.3)	60
Anaesthesiology	1 (25)	4 (100)	4 (100)	1 (25)	1 (25)	3 (75)	0 (0)	3 (75)	0 (0)	3 (75)	0 (0)	4
Child and adolescent psychiatry	2 (28.6)	3 (42.9)	4 (57.1)	2 (28.6)	3 (42.9)	1 (14.3)	0 (0)	3 (42.9)	1 (14.3)	3 (42.9)	0 (0)	7
Gynaecology and obstetrics	0 (0)	2 (22.2)	1 (11.1)	3 (33.3)	4 (44.4)	5 (55.6)	0 (0)	3 (33.3)	0 (0)	5 (55.6)	2 (22.2)	9
Internal medicine	1 (14.3)	2 (28.6)	2 (28.6)	1 (14.3)	1 (14.3)	2 (28.6)	0 (0)	0 (0)	0 (0)	5 (71.4)	2 (28.6)	7
Surgery	1 (50)	0 (0)	2 (100)	0 (0)	0 (0)	1 (50)	0 (0)	0 (0)	0 (0)	2 (100)	0 (0)	2
Orthopaedic surgery	1 (50)	0 (0)	1 (50)	0 (0)	0 (0)	1 (50)	0 (0)	0 (0)	0 (0)	1 (50)	1 (50)	2
Plastic surgery	0 (0)	0 (0)	0 (0)	1 (50)	1 (50)	0 (0)	0 (0)	0 (0)	0 (0)	0 (0)	0 (0)	2
Paediatrics	5 (33.3)	7 (46.7)	11 (73.3)	3 (20)	4 (26.7)	9 (60)	1 (6.7)	7 (46.7)	6 (40)	10 (66.7)	3 (20)	15
Rheumatology	5 (41.7)	6 (50)	7 (58.3)	2 (16.7)	3 (25)	10 (83.3)	2 (16.7)	5 (41.7)	2 (16.7)	9 (75)	4 (33.3)	12
Ophthalmology	1 (7.7)	0 (0)	2 (15.4)	1 (7.7)	2 (15.4)	3 (23.1)	0 (0)	4 (30.8)	0 (0)	4 (30.8)	5 (38.5)	13
Otorhino-laryngology	1 (12.5)	0 (0)	1 (12.5)	0 (0)	1 (12.5)	3 (37.5)	0 (0)	1 (12.5)	0 (0)	0 (0)	5 (62.5)	8

Regarding the suitability of video consultation for a new patient, some psychiatrists noted that for certain patients with diagnoses like anxiety or autism, showing up at the clinic may be too overwhelming. Thus, video consultation enables them to meet their psychiatrist for the first time in safe surroundings. Some specialists commented that simple consultations such as providing test results or adjustments of medication were quicker and easier by telephone than video.

Some specialists reported in the open text fields that video consultation was not suitable for complex symptoms or diagnoses, due to the lack of overall impression of the patient. An ophthalmologist touched upon a holistic approach to the patient: *“I don’t believe that you through a screen can read the more psychological aspects of a patient’s condition. That is, the more unspecific circumstances which are crucial in order to treat “the whole person”. How does the patient act when they enter the clinic/*meeting *with the staff/are they anxious/out of breath, etc. That is, the good old virtues on which medical science is built”.* Aligned with this quotation, *s*everal specialists emphasized that the physical presence of the patients is an important part of the diagnostic picture, and that video consultation blurs this.

### Medical relevancy for the speciality

In total, 47.3% of the respondents did not find video consultation medically relevant to their specialty (Table 3). All child and adolescent psychiatrists found video consultation medically relevant, while no radiologists found it medically relevant. Compared to dermato-venereology, the specialties least likely to offer video consultation due to medical irrelevancy were otorhinolaryngology (OR 30.5, 95% CI 11.3–82.4), ophthalmology (OR 22.7, 95% CI 8.4–61.1) and internal medicine (OR 11, 95% CI 3.4–35.5). Psychiatrists were least likely to consider video consultation medically irrelevant (OR 0.3, 95% 0.05–1.3).

**Table 3:** To what degree the medical specialists will offer video consultation upon patient request. *(N = 499)*

	Not medically relevant	Low degree	Some degree	High degree	Total	OR for not medically relevant *N* = 499	
	n (%)	n (%)	n (%)	n (%)	n (%)	OR (95% CI)	***P***
Specialty							
Total	236 (47.3)	97 (19.4)	72 (14.4)	94 (18.8)	499 (100)		
Dermato-venerology	6 (11.3)	21 (39.6)	18 (34)	8 (15.1)	53 (100)	1	
Neurology	3 (18.8)	1 (6.3)	5 (31.3)	7 (43.8)	16 (100)	1.8 (0.4–8.2)	0.444
Psychiatry	2 (3.1)	11 (16.9)	15 (23.1)	37 (56.9)	65 (100)	0.3 (0.05–1.3)	0.097
Anaesthesiology	8 (57.1)	0 (0)	2 (14.3)	4 (28.6)	14 (100)	10.4 (2.7–40.6)	< 0.001
Child and adolescent psychiatry	0 (0)	2 (28.6)	2 (28.6)	3 (42.9)	7 (100)	*	
Radiology	9 (100)	0 (0)	0 (0)	0 (0)	9 (100)	*	
Gynaecology and obstetrics	20 (43.5)	11 (23.9)	8 (17.4)	7 (15.2)	46 (100)	6 (2.2–16.9)	< 0.001
Internal medicine	14 (58.3)	6 (25)	1 (4.2)	3 (12.5)	24 (100)	11 (3.4–35.5)	< 0.001
Surgery	17 (65.4)	2 (7.7)	2 (7.7)	5 (19.2)	26 (100)	14.8 (4.6–47.8)	< 0.001
Orthopaedic surgery	9 (52.9)	6 (35.3)	1 (5.9)	1 (5.9)	17 (100)	8.8 (2.5–31.6)	< 0.001
Plastic surgery	10 (71.4)	1 (7.1)	1 (7.1)	2 (14.3)	14 (100)	19.6 (4.7–82.5)	< 0.001
Paediatrics	3 (17.6)	3 (17.6)	3 (17.6)	8 (47.1)	17 (100)	1.7 (0.4–7.6)	0.501
Rheumatology	7 (28)	4 (16)	9 (36)	5 (20)	25 (100)	3 (0.9–10.3)	0.073
Ophthalmology	58 (74.4)	13 (16.7)	4 (5.1)	3 (3.8)	78 (100)	22.7 (8.4–61.1)	< 0.001
Otorhinolaryngology	70 (79.5)	16 (18.2)	1 (1.1)	1 (1.1)	88 (100)	30.5 (11.3–82.4)	< 0.001

* Numbers not shown due to all respondents in one category

Of the medical specialists finding video consultation medically irrelevant, 80.9% stated that the *medical issues* were not suitable for video, 59.7% found the *treatment* not suitable for video, that is, treatments requiring the physical presence of the patient, such as inspection and treatment of nose and throat; and 45.7% believed that their *diagnostic ability* was impaired on video. A rheumatologist commented: *“Video consultation cannot be used in my specialty, where all examination and treatment take place in a “hands-on” manner, i.e., through physical contact with the patient, and with diagnostic ultrasound scanning performed by me as a specialist”.* Furthermore, video consultation was considered medically irrelevant due to conversation on video being inadequate (12.2%), problems with sound and picture (11.9%) and the format being too time-consuming (11.4%) (see Additional file 4).

### Trust and care in patient-doctor relationship

Among the respondents who have used video consultation, 55.1% considered the trust and care in the relationship with the patient to be the same during this consultation form as during a physical consultation, whereas 42.2% deemed it worse and 2.6% considered it better.

In cases where a physical consultation had preceded a video consultation, multiple medical specialists highlighted that mutual trust remained present in video consultations. In these situations, the specialists considered themselves able to maintain patient trust and provide the same quality of care that they would in physical consultations. Establishing trust with a new patient was considered difficult when video consultation is the first point of contact. Moreover, some specialists highlighted that communication during video consultations may be deficient, and thus making sure that the patient has understood the instructions was challenging. Some specialists underlined that compliance might be lacking as a result. Furthermore, the intangible ‘feeling’ about the patient, and the personal contact with the patient were not considered the same, and according to some psychiatrists not possible to achieve through this medium. Regarding maintaining trust and care, a psychiatrist commented: *“It depends a lot on the patient. Younger individuals are generally more comfortable with video consultations, and it is easier to build trust with them. However, it can be challenging to make eye contact and establish a strong sense of presence”.* This highlights that successful video consultation depends on and varies among individual patients.

### New attention to time and accessibility

Through the open text, specialists brought the aspects of time and accessibility to our attention. The most highlighted advantage of video consultation was the time saved by patients (transport and taking time off their jobs). Specialists also reported that they manage their own time more effectively when using video consultation, and some mentioned that the consultations were more efficient. An anaesthesiologist commented: “*The consultation has been more focused and more about the patient himself and the problem, as we haven’t used time on: Where to hang my clothes, where is the toilet– so the logistics about the patient is removed. Now they are ready at the time of the appointment and all time is spent on the conversation”.* In line with other comments, this illustrates that the conversation during video becomes more focused and straight to the point. Nevertheless, some specialists stressed that video consultations were more time-consuming as it takes time to set up the technology.

Another important aspect was fewer cancellations. Especially for psychiatrists, where patients might suffer from conditions such as anxiety, making it difficult to show up at a physical consultation. According to the specialists in our study, patients with physical impairment also benefitted from video consultation. Moreover, specialists stressed that they could take in patients from other regions, and furthermore, video consultation makes it easier for relatives from different parts of the country to participate in patient consultations.

## Discussion

The present study demonstrated a relatively low current use of video consultation among medical specialists in private practice in Denmark with the exception of psychiatrists, where more than 80% reported using video consultation. The medical specialists reported that video consultation was most suitable for follow-up consultations and for patients with whom a relationship had already been established in person. Among current users, 79.5% found video consultation as a permanent option in the collective agreement positive. A total of 47.3% of the respondents did not find video consultation medically relevant to their specialty. This was especially predominant among these specialties: radiology, surgery, ophthalmology, and otorhinolaryngology, mostly due to medical issues requiring the physical presence of the patient.

In line with our findings, a German study found that the majority of rheumatologists perceived video consultation most suitable for follow-up visits and not for new patients [27]. The authors conclude that video consultation primarily should be used as a supplement. This also resonates with our findings of most specialists stating that video consultations cannot stand alone but needs to be in combination with physical consultations. Similar to our findings of specialists preferring video consultation with known patients in longer treatment courses, previous studies have underlined the importance of a pre-existing doctor-patient relationship for a successful video consultation [3, 28–30].

In our study, video consultation is mostly used by psychiatrists, which reflects findings from a study conducted in Scotland. The authors found that video consultation was very useful in psychiatry while in specialties where visual examination was crucial, video was unsuitable [29]. Similar results were found in a review of 28 studies on video consultation among health professionals in different clinical settings [9]. Similar to our findings, suitability in specific situations rather than general suitability was emphasized in the review as some conditions are more appropriate to be assessed digitally, for example, mental health issues and simpler skin conditions [9]. Resembling our findings, the Scottish clinicians highlighted improved and faster access to specialist care for the patients as well as video consultation helping patients overcome reluctance to visit the clinic [29].

We find that across different specialities, specialists find their diagnostic abilities impaired on video. This resonates with another Scottish study, which found that most of the included neurologists were not confident about the quality of their neurological assessments when done by video [31], indicating that diagnosing by video consultation may be suboptimal. Our findings also indicate that a physical follow-up consultation is sometimes needed after a video consultation, resulting in additional consultations, putting further pressure on the waiting lists for medical specialists. From a healthcare system perspective, it is therefore relevant to investigate when, for whom and under what circumstances video consultation is useful.

Even though many healthcare systems in Europe push for more digital healthcare, our study underlines that the suitability of video consultation to a large extent depends on the individual patient’s medical condition, the specialty, and the medical specialist’s attitude towards, and experience with, video consultation. Given the technical problems that some specialists experience in setting up and conducting video consultations, it is also crucial to provide the necessary IT support and video consultation training for medical professionals if video consultations are to be successful.

### Strengths and limitations

The strengths of this study include the national coverage of the survey together with a relatively high response rate. Moreover, our study is conducted after COVID-19 was under control in Denmark and therefore it represents use of video consultation not influenced by restrictions hindering physical attendance. The quantitative results were supported by comments in the open text fields, which allowed us to further understand and make sense of the survey data. As no validated survey on this issue exists, we developed, through careful procedures, our own survey suitable for a Danish context. However, the survey was only piloted with one medical specialist and did not undergo psychometric testing, so we cannot rule out validity and reliability issues of the items. The survey may have attracted specialists with stronger opinions– both positive and negative– towards digital solutions and thus we cannot be certain that the sample represents the general use of and opinion on video consultation among medical specialists in Denmark.

The findings underscore that the distinct medical specialties attribute varying importance to visual and tactile modalities, as well as the role of dialogue, in the context of diagnostics and treatment. Consequently, there is significance in examining medical specialists within the framework of their respective specialties. The term ‘medical irrelevancy’ covers a wide variety of reasons. Thus, research into what constitutes ‘medical irrelevancy’ for the different specialties is warranted.

## Conclusion

Uptake of video consultation among medical specialists in private practice in Denmark is not widespread and almost half find it irrelevant for their medical specialty. However, in psychiatry where cancellations are frequent and the conversation plays a major role in the consultation and treatment, specialists report that video consultation is a meaningful tool. In other specialties for patients with chronic diseases, video consultation may be a useful and beneficial supplement to physical consultations.

The political aspiration to digitization in the healthcare system should be rooted in the professionals’ and patients’ perceptions and experiences with video consultation and embrace the fact that it is not a standard tool for all consultations.

### Electronic supplementary material

Below is the link to the electronic supplementary material.

Additional file 1. Survey questionnaire.

Additional file 2. Proportion of video consultations out of all consultations.

Additional file 3. Opinion on video consultation becoming part of the collective agreement in 2022.

Additional file 4. Reasons for video consultation being medically irrelevant.

## Data Availability

The datasets used and/or analysed during the current study are available from the corresponding author on reasonable request.

## References

[1] Haleem A, Javaid M, Singh RP (2021). Telemedicine for healthcare: capabilities, features, barriers, and applications. Sens Int.

[2] Wherton J, Greenhalgh T (2020). Evaluation of the attend Anywhere / Near me video consulting service in Scotland, 2019-20.

[3] Greenhalgh T, Shaw S, Wherton J (2018). Real-world implementation of video outpatient consultations at Macro, Meso, and Micro levels: mixed-method study. J Med Internet Res.

[4] Almathami HKY, Win KT, Vlahu-Gjorgievska E (2020). Barriers and facilitators that influence Telemedicine-Based, Real-Time, Online Consultation at patients’ homes: systematic literature review. J Med Internet Res.

[5] Donaghy E, Atherton H, Hammersley V (2019). Acceptability, benefits, and challenges of video consulting: a qualitative study in primary care. Br J Gen Pract.

[6] Gray C, Wray C, Tisdale R (2022). Factors influencing how Providers assess the appropriateness of video visits: interview study with primary and Specialty Health Care Providers. J Med Internet Res.

[7] Cowan KE, McKean AJ, Gentry MT (2019). Barriers to Use of Telepsychiatry: clinicians as gatekeepers. Mayo Clin Proc.

[8] Due TD, Thorsen T, Andersen JH (2021). Use of alternative consultation forms in Danish general practice in the initial phase of the COVID-19 pandemic– a qualitative study. BMC Fam Pract.

[9] Lampickienė I, Davoody N (2022). Healthcare professionals’ experience of performing Digital Care Visits-A scoping review. Life Basel Switz.

[10] Baluszek JB, Wiig S, Myrnes-Hansen KV (2022). Specialized healthcare practitioners’ challenges in performing video consultations to patients in Nordic Countries– a systematic review and narrative synthesis. BMC Health Serv Res.

[11] Christensen LF, Moller AM, Hansen JP (2020). Patients’ and providers’ experiences with video consultations used in the treatment of older patients with unipolar depression: a systematic review. J Psychiatr Ment Health Nurs.

[12] Gullslett MK, Kristiansen E, Nilsen ER (2021). Therapists’ experience of Video Consultation in Specialized Mental Health services during the COVID-19 pandemic: qualitative interview study. JMIR Hum Factors.

[13] The Danish Health Data Authority. Digital health strategy 2018–2022. Copenhagen: Report; January 2018.

[14] Danish Regions. Danske Regioner - Hver tredje kontakt med lægen skal være digital, https://www.regioner.dk/services/nyheder/2018/oktober/hver-tredje-kontakt-med-laegen-skal-vaere-digital (2018, accessed 15 June 2022).

[15] OECD, European Union. *Health at a Glance: Europe 2022: State of Health in the EU Cycle*. OECD. Epub ahead of print 5 December 2022. 10.1787/507433b0-en.

[16] General Practioners’ Organisation. Min Læge, https://www.laeger.dk/foreninger/plo/digital-praksis/min-laege/ (accessed 15 June 2022).

[17] Danish Health Authority. *COVID-19: Monitorering af aktivitet i sundhedsvæsenet*. Report 2, Copenhagen, 6 July 2020.

[18] Reddy A, Gunnink E, Deeds SA (2020). A rapid mobilization of ‘virtual’ primary care services in response to COVID-19 at Veterans Health Administration. Healthc Amst Neth.

[19] Saint-Lary O, Gautier S, Le Breton J (2020). How GPs adapted their practices and organisations at the beginning of COVID-19 outbreak: a French national observational survey. BMJ Open.

[20] Danish Regions. Danske Regioner - Et år med corona, https://www.regioner.dk/services/nyheder/2021/marts/et-aar-med-corona (2021, accessed 4 July 2023).

[21] The Danish Health Data Authority. Nøgletal for sygehusvæsenet og praksisområdet. *eSundhed*, https://www.esundhed.dk/Emner/Patienter-og-sygehuse/Noegletal-for-sygehusvaesenet-og-praksisomraadet#tabpanel4658D14B214648249412FC1C303829E3 (accessed 31 May 2023).

[22] Birk HO, Vrangbæk K, Rudkjøbing A et al. Denmark: Health system review. In: *Health Systems in Transitions*. 2023.

[23] Foreningen af Speciallæger. Regionernes Lønnings- Og Takstnævn. Overenskomst Om Speciallægehjælp mellem Foreningen Af Speciallæger (FAS) og Regionernes Lønnings- Og Takstnævn (RLTN). Copenhagen: Foreningen af speciallæger; April 2022.

[24] Jessen B. Flere digitale konsultationer– men bruger vi dem rigtigt? *Ugeskriftet.dk*, https://ugeskriftet.dk/nyhed/flere-digitale-konsultationer-men-bruger-vi-dem-rigtigt (2022, accessed 4 July 2023).

[25] Attride-Stirling J. Thematic networks: an analytic tool for qualitative research. In: *Qualitative Research*. 2001, pp. 385–405.

[26] Komitéloven § 14. stk 2. *Danske Love*, https://danskelove.dk/komit%C3%A9loven/14 (accessed 20 January 2024).

[27] Richter JG, Chehab G, Reiter J et al. Evaluation of the use of video consultation in German rheumatology care before and during the COVID-19 pandemic. *Front Med*; 9, https://www.frontiersin.org/articles/10.3389/fmed.2022.1052055 (2022, accessed 13 January 2024). 10.3389/fmed.2022.1052055 PMC9732003 36507506

[28] Assing Hvidt E, Christensen NP, Grønning A (2022). What are patients’ first-time experiences with video consulting? A qualitative interview study in Danish general practice in times of COVID-19. BMJ Open.

[29] Wherton J, Greenhalgh T, Shaw SE (2021). Expanding Video Consultation services at Pace and Scale in Scotland during the COVID-19 pandemic: national mixed methods Case Study. J Med Internet Res.

[30] Johnsen TM, Norberg BL, Kristiansen E (2021). Suitability of Video consultations during the COVID-19 pandemic Lockdown: cross-sectional Survey among Norwegian General practitioners. J Med Internet Res.

[31] Stavrou M, Lioutas E, Lioutas J (2021). Experiences of remote consulting for patients and neurologists during the COVID-19 pandemic in Scotland. BMJ Neurol Open.

