# Supplementary material for: Medical specialists’ use and opinion of video consultation in Denmark: a survey study
Source: BMC Health Serv Res. 2024 Apr 24;24:516. doi: 10.1186/s12913-024-10868-6 (PMC11044495; doi:10.1186/s12913-024-10868-6)
Supplement: Supplementary file 1 — Additional file 1. Survey questionnaire. [file 12913_2024_10868_MOESM1_ESM.docx]

**Additional file 1: Survey questionnaire**

**Q1. What is your medical specialty?**

1. Anaesthesiology
2. Child and adolescent psychiatry
3. Dermato-venerology
4. Radiology
5. Gynaecology and obstetrics
6. Internal medicine
7. Surgery
8. Neurology
9. Orthopaedic surgery
10. Plastic surgery
11. Psychiatry
12. Paediatrics
13. Rheumatology
14. Ophthalmology
15. Otorhinolaryngology
16. Other provider-number

**Q2. From which region do you have a provider-number?**

1. Capital Region of Denmark
2. Central Denmark Region
3. North Denmark Region
4. Region Zealand
5. Region of Southern Denmark
6. No provider-number

**Q3. Have you used or do you use video consultation in your practice?**

1. Yes, both before and during COVID-19 lockdown and I still use it
2. Yes, during COVID-19 lockdown and I still use it
3. Yes, but only during COVID-19 lockdown and I do not use it anymore
4. Yes, after the new collective agreement took effect 1^st^ April 2022
5. No, I have never used it

**Q4. What is your opinion on video consultation becoming part of the collective agreement in 2022? (see excerpt from § 45)**

**§ 45. Video consultation between medical specialist and patient. Video consultation must be offered by all medical specialists in private practice to the extent that it is medically relevant for the specialty. When the medical specialists assesses that it is medically relevant, and the patient requests it, consultation with the patient can take place via video instead in-person (..) (FAS and RTLN, Overenskomst, 2022)**

1. Positive
2. Mostly positive
3. Neutral
4. Mostly negative
5. Negative

**Please elaborate why you are positive/neutral/negative.**

Open text field

**Q5. How many of your consultations in the last month have been by video?**

1. 0-10%
2. 11-20%
3. 21-30%
4. 31-60%
5. 60+%

**Q6. Which type of medium do you use for your video consultations?** (multiple responses possible)

1. MyDoctor
2. Zoom
3. Skype
4. Teams
5. Google Meet
6. FaceTime
7. WhatsApp
8. Other

**Q7. What are your experiences with video consultations?**

1. Very positive
2. Positive
3. Neutral
4. Negative
5. Very negative

**Please elaborate why your experiences are positive/neutral/negative.**

Open text field

**Q8. For which type of consultations do you think video is suitable?** (multiple responses possible)

1. Short treatment courses
2. Long treatment courses
3. With a known patient
4. With a new patient
5. For anamnesis
6. For follow-up of test result(s)
7. Objective assessment
8. For control of medication
9. For treatment
10. For follow-up consultations
11. No types of consultations

**Please elaborate.**

Open text field

**Q9. To what degree would you offer video consultation if the patient requested it?**

1. High degree
2. Some degree
3. Low degree
4. It is now medically relevant for my specialty to do video consultations

**Q10. Who requests in most cases video consultation?**

1. The medical specialist
2. The patient
3. Both request it equally

**Q11. Why is video consultation not medically relevant for your specialty?** (multiple responses possible)

1. The medical issues are not suitable for video
2. The treatment is not suitable for video
3. Problems with sound and picture complicates an optimal consultation
4. Conversation through video complicates an optimal consultation
5. The diagnostic basis is impaired by the video format
6. The IT setup is difficult
7. The format is too time consuming

**Please elaborate.**

Open text field

**Q12. How do you experience care and trust in the relationship with the patient during a video consultation compared to a physical consultation?**

1. Much better
2. Better
3. The same
4. Worse
5. Much worse

**Please elaborate why it is better/the same/worse.**

Open text field
