# Supplementary material for: Medical specialists’ use and opinion of video consultation in Denmark: a survey study
Source: BMC Health Serv Res. 2024 Apr 24;24:516. doi: 10.1186/s12913-024-10868-6 (PMC11044495; doi:10.1186/s12913-024-10868-6)
Supplement: Supplementary file 2 — Additional file 2. Proportion of video consultations out of all consultations. [file 12913_2024_10868_MOESM2_ESM.docx]

**Additional file 2: Proportion of video consultations out of all consultations in a month, by medical specialty. Only current users. *N=117***

|  | 0-10 % | 11-20 % | 21-30 % | 31-60 % | 60+ % | Total |
| --- | --- | --- | --- | --- | --- | --- |
|  | n (%) | n (%) | n (%) | n (%) | n (%) | n (%) |
| Specialty |  | | | | | |
| Total | 82 (70.1) | 21 (17.9) | 9 (7.7) | 2 (1.7) | 3 (2.6) | 117 (100) |
| Dermato-venerology | 14 (9.3) | 1 (6.7) | 0 | 0 | 0 | 15 (100) |
| Neurology | 6 (60) | 3 (30) | 1 (10) | 0 | 0 | 10 (100) |
| Psychiatry | 34 (61.8) | 12 (21.8) | 6 (10.9) | 2 (3.6) | 1 (1.8) | 55 (100) |
| Anaesthesiology | 2 (50) | 0 | 0 | 0 | 2 (50) | 4 (100) |
| Child and adolescent psychiatry | 5 (100) | 0 | 0 | 0 | 0 | 5 (100) |
| Radiology | 0 | 0 | 0 | 0 | 0 | 0 |
| Gynaecology and obstetrics | 4 (66.7) | 2 (33.3) | 0 | 0 | 0 | 6 (100) |
| Internal medicine | 3 (100) | 0 | 0 | 0 | 0 | 3 (100) |
| Surgery | 0 | 0 | 0 | 0 | 0 | 0 |
| Orthopaedic surgery | 1 (100) | 0 | 0 | 0 | 0 | 1 (100) |
| Plastic surgery | 1 (100) | 0 | 0 | 0 | 0 | 1 (100) |
| Paediatrics | 5 (55.6) | 2 (22.2) | 2 (22.2) | 0 | 0 | 9 (100) |
| Rheumatology | 5 (83.3) | 1 (16.7) | 0 | 0 | 0 | 6 (100) |
| Ophthalmology | 2 (100) | 0 | 0 | 0 | 0 | 2 (100) |
| Otorhinolaryngology | 0 | 0 | 0 | 0 | 0 | 0 |
