# Supplementary material for: Medical specialists’ use and opinion of video consultation in Denmark: a survey study
Source: BMC Health Serv Res. 2024 Apr 24;24:516. doi: 10.1186/s12913-024-10868-6 (PMC11044495; doi:10.1186/s12913-024-10868-6)
Supplement: Supplementary file 3 — Additional file 3. Opinion on video consultation becoming part of the collective agreement in 2022. [file 12913_2024_10868_MOESM3_ESM.docx]

|  | **Positive** | **Neutral** | **Negative** | **Total** |
| --- | --- | --- | --- | --- |
|  | n (%) | n (%) | n (%) | n (%) |
| **Not current user** | 87 (22.8) | 125 (32.7) | 170 (44.5) | 382 (100) |
| **Current user** | 93 (79.5) | 18 (15.4) | 6 (5.1) | 117 (100) |

**Additional file 3: Opinion on video consultation becoming part of the collective agreement in 2022. *N=499***

*P<0.001. Differences between groups tested with a chi-square test.*
