# Supplementary material for: Medical specialists’ use and opinion of video consultation in Denmark: a survey study
Source: BMC Health Serv Res. 2024 Apr 24;24:516. doi: 10.1186/s12913-024-10868-6 (PMC11044495; doi:10.1186/s12913-024-10868-6)
Supplement: Supplementary file 4 — Additional file 4. Reasons for video consultation being medically irrelevant. [file 12913_2024_10868_MOESM4_ESM.docx]

**Additional file 4: Reasons for video consultation being medically irrelevant, by medical specialists. Only medical specialists who do not find video consultation medically relevant. Multiple responses possible. *N=236***

|  | **Medical issues not suitable for video** | **Treatment not suitable for video** | **Problems with sound and picture impedes an optimal difficult** | **Conversation via video impedes an optimal consultation** | **Diagnostic ability is impaired because of the video format** | **IT setup is troublesome** | **The format is too time-consuming** | **Total respondents** |
| --- | --- | --- | --- | --- | --- | --- | --- | --- |
|  | **n (%)** | **n (%)** | **n (%)** | **n (%)** | **n (%)** | **n (%)** | **n (%)** | **n (%)** |
| **Specialty** |  |  |  |  |  |  |  |  |
| **Total** | 191 (80.9) | 141 (59.7) | 28 (11.9) | 29 (12.2) | 108 (45.7) | 11 (4.6) | 27 (11.4) | 236 (100) |
| **Dermato-venerology** | 4 (66.7) | 3 (50) | 2 (33.3) | 2 (33.3) | 5 (83.5) | 0 | 2 (33.3) | 6 (100) |
| **Neurology** | 0 | 0 | 2 (66.7) | 0 | 1 (33.3) | 2 (66.7) | 2 (66.7) | 3 (100) |
| **Psychiatry** | 1 (50) | 1 (50) | 0 | 2 (100) | 1 (50) | 0 | 0 | 2 (100) |
| **Anaesthesiology** | 5 (62.5) | 6 (75) | 0 | 1 (12.5) | 0 | 0 | 0 | 8 (100) |
| **Radiology** | 9 (100) | 5 (55.6) | 0 | 0 | 0 | 0 | 0 | 9 (100) |
| **Gynaecology and obstetrics** | 18 (90) | 13 (65) | 0 | 1 (5) | 1 (5) | 0 | 1 (5) | 20 (100) |
| **Internal medicine** | 9 (64.3) | 4 (28.6) | 4 (28.6) | 2 (14.3) | 8 (57.1) | 2 (14.3) | 4 (28.6) | 14 (100) |
| **Surgery** | 12 (70.6) | 11 (64.7) | 2 (11.8) | 3 (17.6) | 6 (35.3) | 0 | 0 | 17 (100) |
| **Orthopaedic surgery** | 7 (77.8) | 5 (55.6) | 0 | 2 (22.2) | 3 (33.3) | 0 | 1 (11.1) | 9 (100) |
| **Plastic surgery** | 9 (90) | 8 (80) | 1 (10) | 0 | 4 (40) | 0 | 0 | 10 (100) |
| **Paediatrics** | 2 (66.7) | 1 (33.3) | 0 | 1 (33.3) | 2 (66.7) | 0 | 1 (33.3) | 3 (100) |
| **Rheumatology** | 6 (85.7) | 6 (85.7) | 1 (14.3) | 0 | 4 (57.1) | 0 | 0 | 7 (100) |
| **Ophthalmology** | 49 (84.5) | 32 (55.2) | 7 (12.1) | 5 (8.6) | 37 (63.8) | 4 (6.9) | 9 (15.5) | 58 (100) |
| **Otorhinolaryngology** | 60 (85.7) | 46 (65.7) | 9 (12.9) | 10 (14.3) | 36 (51.4) | 3 (4.3) | 7 (10) | 70 (100) |
